# Correction: Urtica dioica Agglutinin: A plant protein candidate for inhibition of SARS-COV-2 receptor-binding domain for control of Covid19 Infection

**DOI:** 10.1371/journal.pone.0314614

**Published:** 2024-11-22

**Authors:** Fatemeh Sabzian-Molaei, Mohammad Ali Nasiri Khalili, Mohammad Sabzian-Molaei, Hosein Shahsavarani, Alireza Fattah Pour, Ahmad Molaei Rad, Amin Hadi

There are errors in the Author Contributions. The correct contributions are:

**Formal analysis:** Fatemeh Sabzian-Molaei.

**Investigation:** Fatemeh Sabzian-Molaei, Mohammad Ali Nasiri Khalili, Mohammad Sabzian-Molaei.

**Methodology:** Fatemeh Sabzian-Molaei, Mohammad Ali Nasiri Khalili, Mohammad Sabzian-Molaei, Hosein Shahsavarani, Alireza Fattah Pour, Ahmad Molaei Rad, Amin Hadi.

**Project administration:** Mohammad Ali Nasiri Khalili.

**Software:** Fatemeh Sabzian-Molaei.

**Supervision**: Mohammad Ali Nasiri Khalili.

**Validation**: Fatemeh Sabzian-Molaei, Mohammad Ali Nasiri Khalili, Hosein Shahsavarani.

**Visualization**: Fatemeh Sabzian-Molaei.

**Writing—original draft**: Fatemeh Sabzian-Molaei.

**Writing—review & editing**: Fatemeh Sabzian-Molaei, Mohammad Ali Nasiri Khalili, Amin Hadi.
