# Supplementary material for: Urtica dioica Agglutinin: A plant protein candidate for inhibition of SARS-COV-2 receptor-binding domain for control of Covid19 Infection
Source: PLoS One. 2022 Jul 28;17(7):e0268156. doi: 10.1371/journal.pone.0268156 (PMC9333307; doi:10.1371/journal.pone.0268156)
Supplement: Attachment — Submitted filename: Respose to reviewers.docx [file pone.0268156.s003.docx]

**Dear Editor-in-Chief of PLOS ONE**

I cordially appreciate your reviewers for giving the best comments in order to upgrade our paper. I believe that our paper have significantly improved with these comments. Anyway, all the reviewers' comments have perfectly done which are responded as follows:

Best regards

Amin Hadi

Reviewer 1

Minor

Query 1

Since the mmpbsa calculations were made from the entire MD trajectory, the Table 4 should include standard deviation (S.D +/-) values along with the energy values.

Response

This information is added in Table 4.

**Table 4.** Binding free energies from MM-PBSA calculation.

| **Type of Energeis** | **(kJ/mol)** |
| --- | --- |
| ΔE van der Waals | -333.523 +/- 29.347 |
| ΔE electrostatic | -187.920 +/- 32.598 |
| ΔE Polar solvation | 508.793 +/- 108.926 |
| ΔE SASA | -34.855 +/- 2.174 |
| ΔG _total (_Binding energy) | -47.505 +/- 12.401 |

Query 2

The authors may provide the best docked structure (PDB co-ordinates) available in the supplementary file. (not mandatory)

Response

The best-docked structure (PDB format) is presented in the Supplementary Material.

Thank you for your comments and suggestions.
